# Intelligent ankle–foot prosthesis based on human structure and motion bionics

**DOI:** 10.1186/s12984-024-01414-w

**Published:** 2024-07-13

**Authors:** Baoyu Li, Guanghua Xu, Zhicheng Teng, Dan Luo, Jinju Pei, Ruiquan Chen, Sicong Zhang

**Affiliations:** 1 School of Mechanical Engineering, Xi’an Jiaotong University, Xi’an, 710049 China; 2 State Key Laboratory for Manufacturing Systems Engineering, Xi’an Jiaotong University, Xi’an, 710049 China; 3 The First Affiliated Hospital of Xi’an Jiaotong University, Xi’an, China

**Keywords:** Mechanism design, Rolling conjugate joint, Carbon fiber foot, Rehabilitation prosthesis

## Abstract

The ankle–foot prosthesis aims to compensate for the missing motor functions by fitting the motion characteristics of the human ankle, which contributes to enabling the lower-limb amputees to take care of themselves and improve mobility in daily life. To address the problems of poor bionic motion of the ankle–foot prosthesis and the lack of natural interaction among the patient, prosthesis, and the environment, we developed a complex reverse-rolling conjugate joint based on the human ankle–foot structure and motion characteristics, the rolling joint was used to simulate the rolling-sliding characteristics of the knee joint. Meanwhile, we established a segmental dynamics model of the prosthesis in the stance phase, and the prosthetic structure parameters were obtained with the optimal prosthetic structure dimensions and driving force. In addition, a carbon fiber energy-storage foot was designed based on the human foot profile, and the dynamic response of its elastic strain energy at different thicknesses was simulated and analyzed. Finally, we integrated a bionic ankle–foot prosthesis and experiments were conducted to verify the bionic nature of the prosthetic joint motion and the energy-storage characteristics of the carbon fiber prosthetic foot. The proposed ankle–foot prosthesis provides ambulation support to assist amputees in returning to social life normally and has the potential to help improve clinical viability to reduce medical rehabilitation costs.

## Introduction

The number of individuals with lower limb amputations is increasing [1], which can be caused by natural factors such as disease and traffic accidents and external events such as traffic accidents and war [2, 3]. Active prostheses enable amputees to walk with a more natural gait while simultaneously allowing the users to walk more efficiently on different terrain [4, 5]. A large quantity of lower limb amputees and the advantages of active prostheses motivate researchers to concentrate on the exploration of powered prostheses [6]. It is of great importance to develop a high-performance ankle–foot prosthesis that can realize, as accurately as possible, the bionic function of the human ankle and foot.

The human ankle and foot play a crucial role in enabling them to walk with a natural gait [7]. Because of that, the ankle–foot prosthesis must be highly adaptable to the irregular ground and the motion torque [8, 9]. To achieve this goal, our research focuses on the prosthetic ankle joint's flexibility and the prosthetic foot's ability to absorb ground impact during gait.

The existing ankle prostheses mostly use static joints, fixed-axis rotating joints, and multi-link mechanisms [2]. Static joints are the simplest structure, which compensates for the missing performance of the joints through excellent material properties [10, 11]. Their compensatory capacity is limited, and cannot fully meet the requirements of the ankle motion flexibility of the prosthesis. The fixed-axis rotating joints are more flexible and can fully realize the pitching motion of the joints [11], but they cannot accurately fit the rolling-sliding motion characteristics of the human ankle–foot joint. The trajectory fitting capability of the simple four-link mechanism is limited and becomes worse as the trajectory complexity increases [12]. Increasing the number of links leads to a complex mechanism, which shows the multi-link mechanism is not suitable for the ankle–foot prosthetic [13, 14].

Motivated by the aforementioned shortcomings, this paper is the first step toward bionic design and energy-storage characteristics for prosthetic mobility. The objective is to explore a new prosthetic structure for closely imitating the movement function of the missing biological limb, fitting the roll-slip motion characteristics of the human ankle joint by complex rolling trajectory, which contributes to guaranteeing a locomotion metabolic economy [15, 16].

We focused on the design of rolling conjugate joints and the carbon fiber energy-storage foot's efficient energy storage/release characteristics. Designed to simulate the energy storage and release process of the human foot, to achieve the energy storage when the prosthetic foot is on the ground and the energy released when it leaves the ground, reducing energy consumption. The research works on closely imitating the bionic function of the missing limb to satisfy further requirements provided for lower limb amputees to develop their day-to-day activities, such as walking and climbing stairs [12, 17].

In this article, we show how a bionic design can enable a powered ankle–foot prosthesis to fit within the anatomical foot profile while providing rolling-sliding motion and energy consumption imitation. In the following chapter, the proposed method is discussed in detail. The methodology of the paper starts from Section II on the motion characteristics consisting of brief information on the human gait cycle, conceptual design, actuation, and mechanical structure. Section III describes the dynamic simulations of prostheses for typical locomotion modes and the kinematic and kinetic models for parameter selections of lower limb prostheses. Moreover, it presents the integration of the manufactured prosthesis prototype. Section IV highlights experimental validation, and the experimental results are presented to evaluate the performance of the design. Finally, the key conclusion is presented in Section V, in addition, the limitations of this study and the future research are discussed. The outcome of this article could promote the future design of artificial legs.

## Methodology

The primary design objective of an ankle–foot prosthesis is to provide functional mobility as accurately as possible for common ambulation activities [18]. To design a high-performance ankle–foot prosthesis scientifically, we analyzed the physiological structure of the human foot and ankle [19]. Besides, we explored the motion property of the ankle joint through experiments and summarized its joint angle and joint output power characteristics in the process of movement. Based on this, the overall bionic optimization design of the ankle–foot prosthesis is proposed.

### Extraction of motion characteristics

To analyze joint motion angles, power, and other parameters reflecting the motion characteristics of the human foot and ankle, we collected motion data from the subject's joints by the VICON motion capture system. To investigate a universal design methodology that can be tailored for practical applications according to the physical condition of amputees or individuals with comparable physical attributes. Consequently, the participants in this research are healthy individuals without a history of lower limb trauma in the preceding six months, nor any back, pelvic, neuromuscular disorders, or balance issues. The subjects wore special straitjackets, and a total of 8 markers were pasted on their anterior superior iliac spine, posterior superior iliac spine, thigh, knee, calf, ankle, toe, and heel of the lower limbs. Experiments with two healthy subjects started walking at one end of the force platform, stopped and turned around at the other end, repeated the previous action, and returned to the initial starting point to end the single experiment. The above experimental process was repeated 10 times to get the subjects' movement data respectively in the sagittal plane, including ankle pitching range of motion, joint angle, angular velocity, angular acceleration, and foot–ground contact force. In the ankle range of motion test, the subjects stood on one foot with the hand holding a fixed object, and the test foot was suspended in the air for pitching motion, and the motion was repeated six times in each test, for a total of three tests.

**Fig. 1:**
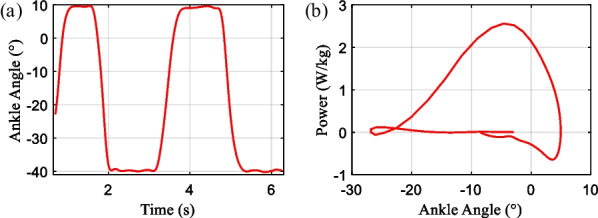
The range of ankle joint angle and its output power

Research shows that human joint angle and power change patterns are similar within a single gait cycle [20, 21]. After data processing, the pitch motion range of the human ankle joint in the sagittal plane is obtained. The experimentally measured ankle joint angle and output power curves are shown in Fig. 1, which is derived from the average of the experimental data from 2 subjects, whose specific information about the subjects is shown in Table 1.

**Table 1:** The sheet of subjects’ information

Parameter	Subject 1	Subject 2
Height (mm)	1801	1730
Weight (kg)	80.6	60.3
Leg length (mm)	945	898
Knee width (mm)	125.5	101.5
Ankle width (mm)	69.5	67.5

### Conceptual design

**Fig. 2:**
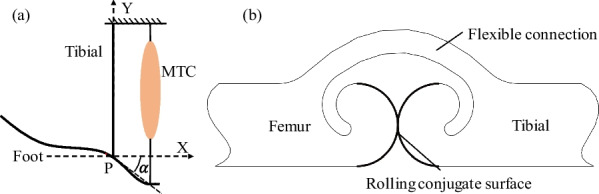
Conceptual design. **a** Simplified ankle–foot model and **b** Symmetrical conjugated trajectories of the rolling joint

A simplified ankle–foot model can be obtained, as shown in Fig. 2a, from the analysis of the physiological characteristics of the healthy human. The movement of the ankle–foot joint is achieved by the tension and contraction of the muscle module (MTC), which changes the angle between the foot and the tibia [22]. The bouncing joints of locusts and crickets are a pair of rigid joints articulated in a specific curved shape and the flexible tissues that connect them. The biological joints are guided by stable motion through the meshing motion of the conjugate surfaces; the flexible tissue ensures that the joints are tightly connected, and at the same time provides the joints with a huge amount of bouncing energy through the stored energy, which generated by the deformation of the flexible tissues.

We proposed a bionic joint model based on exploring the typical insect bouncing joint structure, which is similar to the human ankle joint [23, 24]. It is placed between the tibia and talus to achieve the bionic motion function of the human ankle joint (Fig. 2a). The rolling conjugate surface (Fig. 2b) determines the change in the trajectory of the joint output, which is key to the presented bionic joint in the next section.

### Actuation and mechanical structure

**Fig. 3:**
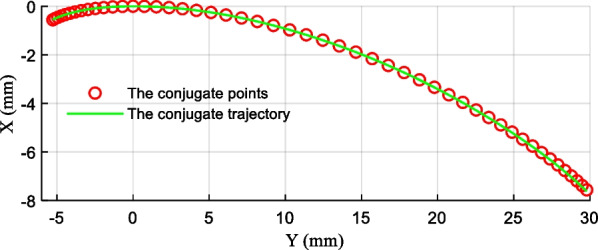
The fitting conjugate trajectory of the rolling joint

Previous studies have shown that the motion of the ankle joint is a combination of rolling and sliding, and its trajectory is in the shape of a “J” curve [25]. We extracted 50 ankle joint data points from the experimental data for analysis, and the conjugate trajectory of the rolling joint can be obtained by transforming the data according to (1). Figure 3 shows the conjugate trajectory. Accordingly, the rolling prosthetic ankle joint model is designed with characteristics of rolling and slipping. The advanced actuation solution is beneficial to the development of the ankle–foot prosthesis available.

1 $$\begin{gathered} {\text{x}}^{\prime}{\text{ = x}} \cdot {\text{cos}}\left( \theta \right) + {\text{y}} \cdot {\text{sin}}\left( \theta \right) \hfill \\ {\text{y}}^{\prime}{\text{ = y}} \cdot {\text{cos}}\left( \theta \right) - {\text{x}} \cdot {\text{sin}}\left( \theta \right) \hfill \\ \end{gathered}$$

where $\theta$ is the rotation angle of the ankle joint, $\left( {x,y} \right)$ is the coordinates of the ankle joint experimental trajectory, $\left( {x^{\prime},y^{\prime}} \right)$ is the coordinates of the rolling joint conjugate trajectory.

In this paper, the three-dimensional reduced integration cell C3D8R is used. the number of meshing of the prosthetic foot is 5184, 3434, 3656, and 3586 for the thickness of 8 mm, 5 mm, 3 mm, and 2 mm respectively. The composite material involved in this model is itself a nonlinear deformation problem, to ensure the accuracy of the model simulation calculation, Explicit Solver is selected in the simulation.

**Fig. 4:**
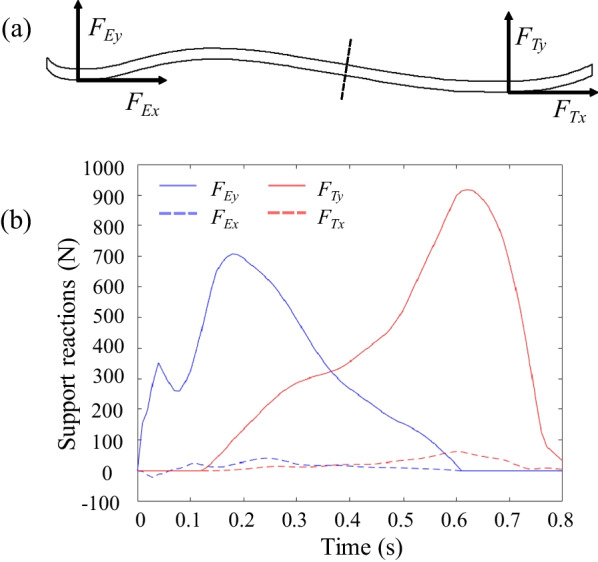
**a** Positional distribution of load loading and its direction **b** The magnitude of the applied load and its distribution

The prosthetic foot is connected to the talus-shaped parts through two bolt holes, so the model is constrained by six degrees of freedom for the two bolt holes. The horizontal and vertical force loads are applied at the lowest position of the toe and heel respectively, and the direction of the load and the point of action are shown in Fig. 4a. The load data are taken from the conversion results of the data from the experimental force measuring table, and the load magnitude changes over time, as shown in Fig. 4b.

The arch height and arc of the energy-storage foot were determined based on the shape of the human foot elastic arch. The finite element simulation analysis provided its thickness selection, presented in Table 2. The characteristics of the energy-storage foot are based on the excellent material properties of carbon fiber, when it is subjected to a large external force impact, it will quickly disperse the force to all parts of the fiber [26]. It is equivalent to adding elastomer at the end of the actuator, which can effectively reduce the flutter and improve the stability of the ankle–foot prosthesis.

**Table 2:** Peak stress on the different thicknesses of prosthetic foot

Thickness (mm)	2	3	5	8
Peak stress (MPa)	9299	874.1	332.1	145.1

The simulation results present that the stress peak of the 2 mm thickness far exceeds the permissible strength of the material. The prosthetic foot with a thickness of 3 mm has a peak stress of 874.1 MPa, which can ensure the requirements.

**Fig. 5:**
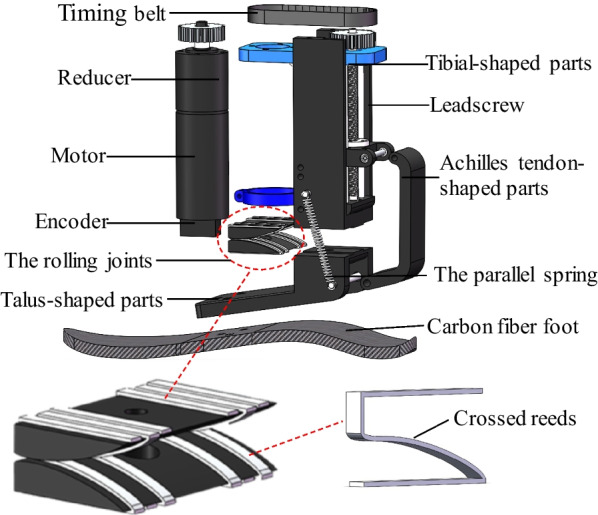
The main components of the developed prosthesis

With the two bionic components, the rolling joint model and the carbon fiber energy-storage foot, as the core, the actuation and mechanical structure of the ankle–foot prosthesis is obtained, as described in Fig. 5. The two bionic components described can be customized according to the amputee's body parameters. The specific trajectory shape of the rolling joint model is the conjugate surface, and the connection between its support blocks is realized by crossed reeds made of stainless steel [27], which is shown in Fig. 5. Two sets of crossed reeds are arranged symmetrically to the left and right, and each set of them consists of three, which play an accurate limiting role through the crossed connection so that the joint always performs movement along the conjugate trajectory and no abnormalities such as sliding occur. Furthermore, we have implemented mechanical and motor electrical limitations on the lead screw to minimize the potential for mechanical failure points, which help prevent user injuries.

The developed prosthesis is an active device driven by the motor that enables amputees to walk with a more natural gait. A timing belt contributes to driving the ball screw and a screw nut leads the Achilles tendon-shaped parts in a reciprocating motion along the axial direction of the screw. The talus-shaped parts are connected by the Achilles tendon-shaped parts and roll along the conjugate surfaces of the rolling joints, and the carbon fiber energy-storage foot is bolted to the talus-shaped parts to simulate human foot motion.

## Dynamic simulations

To meet the motion requirements of the rolling conjugate joint, a kinematic model of the prosthetic mechanism is established, and the kinematic characteristics of the rolling joint fitting of the mechanism under typical gait patterns are analyzed. Meanwhile, a segmental kinetic model of the prosthetic mechanism is established during the gait support phase. Finally, the selection of structural parameters was realized, which aimed to satisfy further requirements such as build height, range of motion, and weight.

**Fig. 6:**
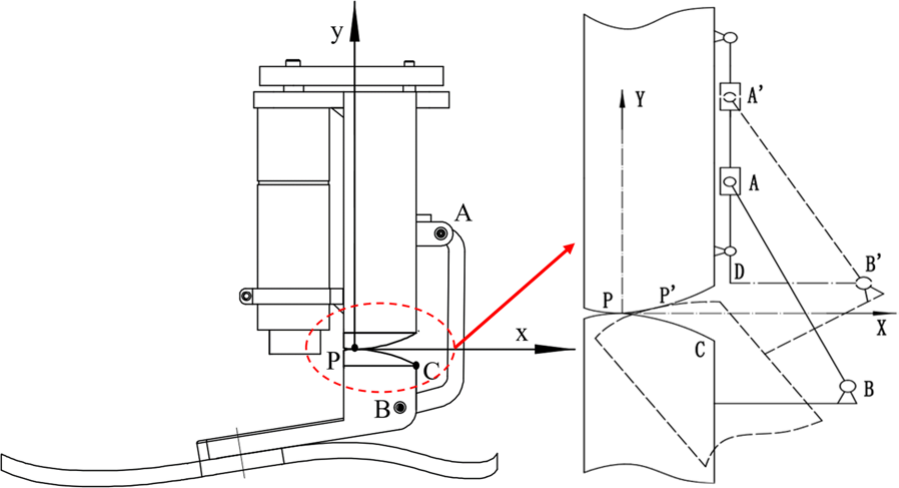
A structural schematic diagram of the ankle–foot prosthesis

Specifically, Fig. 6 depicts a coordinate system, which is established with the conjugate point of the prosthesis in the initial state as the origin. The position and velocity of the key points of the prosthesis are analyzed in this coordinate system. Point P is the instantaneous rolling conjugate point during joint movement. A and B are the hinge points, and C is the conjugate point when the ankle plantar flexion reaches the maximum angle.

### Kinematic model

The talus-shaped parts are always in motion relative to the tibial-shaped parts, so assuming that the tibia is in a fixed state, the tibia is used as a reference position to analyze the movement of the joint output. The solution of the position coordinates based on the rolling joint can be regarded as a motion process of rotation and then translation. The motion-related position points in the prosthetic mechanism mainly include P, C, B, and A. According to its position relationship, the position calculation formula of the key points can be obtained as follows.

2 $$\left\{ \begin{gathered} x_{p} = x_{i} \hfill \\ y_{p} = - y_{i} \hfill \\ \end{gathered} \right.$$

3 $$\left\{ \begin{gathered} x_{i}^{\prime } = \sqrt {x_{i}^{2} + y_{i}^{2} } \cdot \cos \left( {2\alpha_{i} + \arctan \left( {{{y_{i} } \mathord{\left/ {\vphantom {{y_{i} } {x_{i} }}} \right. \kern-0pt} {x_{i} }}} \right)} \right) \hfill \\ y_{i}^{\prime } = \sqrt {x_{i}^{2} + y_{i}^{2} } \cdot \sin \left( {2\alpha_{i} + \arctan \left( {{{y_{i} } \mathord{\left/ {\vphantom {{y_{i} } {x_{i} }}} \right. \kern-0pt} {x_{i} }}} \right)} \right) \hfill \\ \end{gathered} \right.$$

4 $$\left\{ \begin{gathered} x_{A} = a_{1} + d \hfill \\ y_{A} = y_{B} + \sqrt {h^{2} - \left( {x_{B} - x_{A} } \right)^{2} } \hfill \\ \end{gathered} \right.$$

5 $$\left\{ \begin{gathered} x_{B} = \sqrt {\left( {a_{1}^{2} + a_{2}^{2} } \right) + \left( {a_{1}^{2} + a_{2}^{2} } \right)} \cdot \cos \left( {2\alpha_{i} + \arctan \frac{{b_{1} + b_{2} }}{{a_{1} + a_{2} }}} \right) + x_{p} - x_{i}^{\prime } \hfill \\ y_{B} = \sqrt {\left( {a_{1}^{2} + a_{2}^{2} } \right) + \left( {a_{1}^{2} + a_{2}^{2} } \right)} \cdot \sin \left( {2\alpha_{i} + \arctan \frac{{b_{1} + b_{2} }}{{a_{1} + a_{2} }}} \right) + y_{p} - y_{i}^{\prime } \hfill \\ \end{gathered} \right.$$

Define the parameters as follows, $\left( {a_{1} ,b_{1} } \right)$ is the initial coordinates of point C, $\left( {a_{2} ,b_{2} } \right)$ is the initial coordinates of point B, $\left( {x_{p} ,y_{p} } \right)$ is the location of conjugate point P, and the location of point A and B respectively,$\alpha_{i}$ is the rotation angle of the joint, $\left( {x_{i}^{\prime } ,y_{i}^{\prime } } \right)$ is the location conjugate point P at the rotation angle $\alpha_{i}$, the length of the AB connection is h, and d is the lateral distance between the ball screw axis and point C.

The longitudinal displacement of point A in the ankle–foot prosthesis concerning time is the linear velocity of the ball screw nut, so the relationship shown in (6) and (7) is satisfied. Where $\omega$ is the angular velocity of the screw, $S$ denotes the lead of the screw, $N_{s}$ is the speed of the screw, $V_{M}$ is the motor speed, $i_{i}$ is the ratio of the belt, and $i_{M}$ is the motor speed reduction ratio. Here,

6 $$\left\{ \begin{gathered} \frac{{dy_{{\text{A}}} }}{dt} = \frac{\omega }{2\pi }{\text{S}} \hfill \\ {\text{ N}}_{{\text{S}}} = \frac{\omega }{2\pi } \hfill \\ \end{gathered} \right.$$

**Fig. 7:**
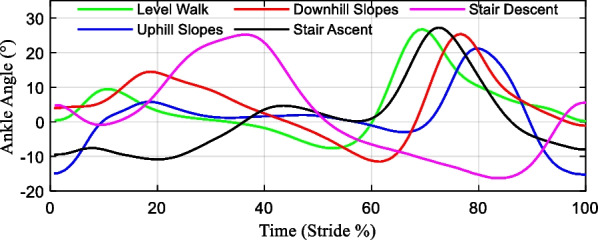
The periodic ankle angle changes of different locomotion modes

As seen in Fig. 7, the periodic ankle angle change curves of different locomotion modes can be obtained through the human gait experiment, which was used as the basis to analyze prosthetic mechanism movements. An analytical model was built to solve the movement position and velocity changes of the developed prosthesis at various joint angles.

The slope of the ground allows the human ankle pitch angle adjusted [28]. Compared with the level walking tasks, the ankle dorsiflexion angle increases and the plantar flexion angle decreases during the uphill slopes, which is the opposite of the downhill process. The approach maintains the balance of the center of gravity. When ascending stairs, the ankle joint mainly maintains balance during the lifting of the center of gravity through plantar flexion. When descending stairs, the ankle joint mainly uses dorsiflexion to maintain balance during the decline of the center of weight.

**Fig. 8:**
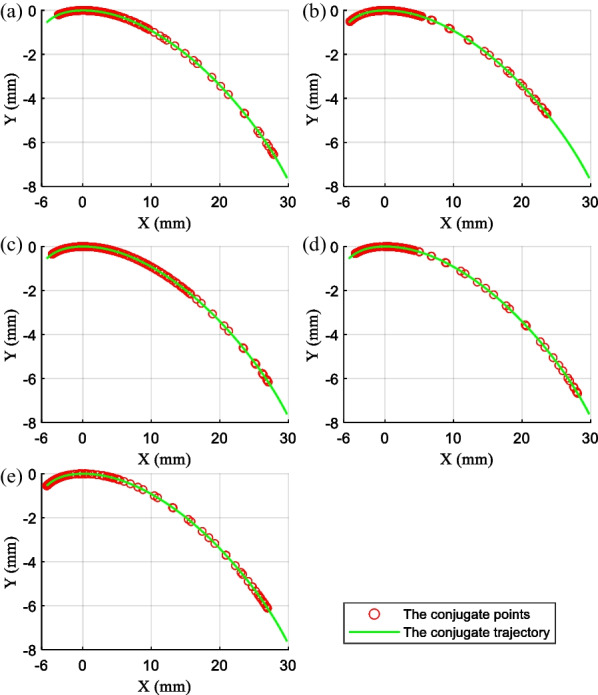
The distribution of conjugate points under typical ambulation patterns. **a** The level walk task. **b** The uphill slopes task. **c** The downhill slopes task. **d** The stair ascent task. **e** The stair descent task

Figure 8 presents the position changes of the rolling conjugate points, which are of the developed ankle–foot prosthesis for typical locomotion tasks, such as walking on a level and inclined ground and climbing stairs. It is demonstrated that the distribution of conjugate points near the initial state is concentrated abnormally. The distribution of them in the different locomotion modes is within the designed trajectory range, which is proof of its practicality preliminary.

7 $$\left\{ \begin{gathered} N_{s} = \frac{1}{S} \cdot \frac{{d\alpha_{{\text{i}}} }}{dt} \cdot \frac{{dy_{A} }}{{d\alpha_{{\text{i}}} }} \hfill \\ V_{M} = N_{s} \cdot i_{t} \cdot i_{M} \hfill \\ \end{gathered} \right.$$

**Fig. 9:**
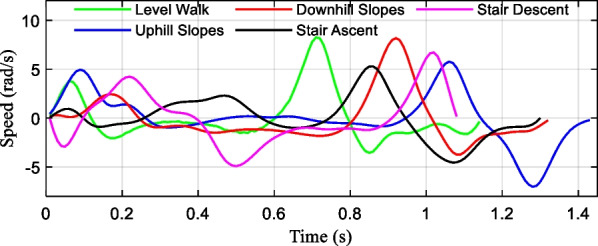
The lead screw speed of different locomotion modes

The lead screw speed of different locomotion modes in the gait cycle is depicted in Fig. 9. The peak speed required to provide for different locomotion tasks can be concluded. The simulation analysis demonstrated that the difference between the up-down slope process and the flat walking process is that the ankle joint movement angle is offset to the dorsiflexor side or plantar flexion side, and its movement mode has not changed significantly. In the actual movement of the prosthesis, lower-limb amputees can rely on the adjustment of the knee angle to adapt to the environmental road conditions such as small angle slopes. In the process of stair ascent and stair descent, the key concern is lifting or lowering the center of gravity, so the process is quite different from that of walking on level and inclined ground.

In this section, the typical locomotion modes of the ankle prosthesis are summarized, and the distribution of rolling joint angles is obtained. Integrating the rotational speed of the ball screw during the typical gait cycle, it can be found that the maximum rotational speed is 8.24 rad/s, which occurs during walking on flat ground, and the overall rotational speed of the stair climbing process is lower, with a peak speed of 5.29 rad/s.

### Kinetic model

The position and velocity changes at a certain point in the mechanism can be dynamically analyzed based on the mathematical kinematic model. However, in the process of mechanism movement, it will be subjected to various forces, including external forces and internal forces, so it is necessary to analyze the dynamic response of the mechanism under various force situations[29, 30]. The structural parameters of the prosthetics can be optimized, which aims to tune the dynamic response of the system by combining kinematics and kinetics analysis.

**Fig. 10:**
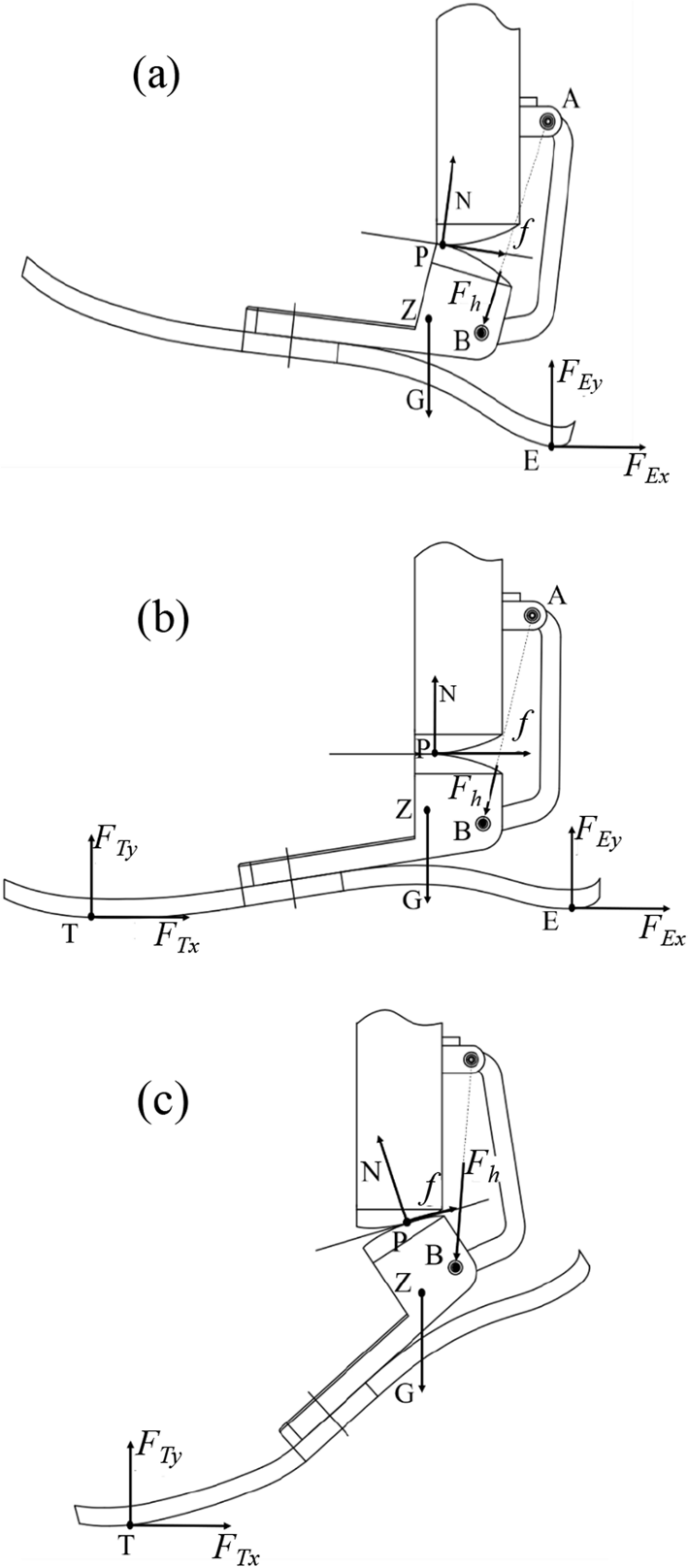
Diagram of ankle–foot prosthesis force. **a** The heel strike phase. **b** The foot-flat phase. **c** The toe-off phase

According to the ground support reaction force, it can be divided into three force states as follows, the heel strike phase, the state of the foot flat, and the toe-off phase [31]. In this section, three dynamic analysis models of prostheses are established. The force analysis is shown in Fig. 10a–c.

In the heel strike phase, the rolling conjugate point P is used as the center of rotation (Fig. 2a), satisfying the equilibrium relationship shown in (8). In the state of the foot flat, its force case satisfies the relationship shown in Eq. (10). In the toe-off phase, the moment balance relationship is shown in (11).

8 $$\left\{ \begin{gathered} F_{h} \cdot d_{h} + G \cdot \left( {x_{Z} - x_{P} } \right) = F_{Ex} \cdot \left( {y_{P} - y_{E} } \right) + F_{Ey} \cdot \left( {x_{E} - x_{P} } \right) + J\ddot{\alpha }_{i} \hfill \\ C_{d} \omega + \vec{F}_{h} \cdot \vec{V}_{h} = 0 \hfill \\ \end{gathered} \right.$$

9 $$d_{h} = \frac{{\left| {\left( {y_{A} - y_{B} } \right) \cdot x_{P} + \left( {x_{B} - x_{A} } \right) \cdot y_{P} + x_{A} \cdot y_{B} - y_{A} \cdot x_{B} } \right|}}{{\sqrt {\left( {y_{A} - y_{B} } \right)^{2} + \left( {x_{B} - x_{A} } \right)^{2} } }}$$

10 $$\left\{ \begin{gathered} F_{h} \cdot d_{h} + G \cdot \left( {x_{Z} - x_{P} } \right) = F_{Ex} \cdot \left( {y_{P} - y_{E} } \right) + F_{Ey} \cdot \left( {x_{E} - x_{P} } \right) + J\ddot{\alpha }_{i} \hfill \\ - F_{Ey} \cdot \left( {x_{E} - x_{P} } \right) - F_{Ex} \cdot \left( {y_{P} - y_{E} } \right) = J\ddot{\alpha }_{i} \hfill \\ C_{d} \omega \pm \vec{F}_{h} \cdot \vec{V}_{h} = 0 \hfill \\ \end{gathered} \right.$$

11 $$\left\{ \begin{gathered} F_{h} \cdot d_{h} - F_{Tx} \cdot \left( {y_{P} - y_{T} } \right) + F_{Ty} \cdot \left( {x_{P} - x_{T} } \right) + + G \cdot \left( {x_{Z} - x_{P} } \right) = J\ddot{\alpha }_{i} \hfill \\ C_{d} \omega \pm \vec{F}_{h} \cdot \vec{V}_{h} = 0 \hfill \\ \end{gathered} \right.$$

Here, $F_{h}$ is the axial tension of connecting rod AB, $\left( {x_{Z} ,y_{Z} } \right)$ is the gravity center of the talus-shaped parts,$G$ is the gravity of the talus-shaped parts, $\left( {x_{E} ,y_{E} } \right)$ is the location of point E, $V_{h}$ is the instantaneous velocity of point B, $C_{d}$ the torque of the ball screw. $d_{h}$ is the vertical distance from point P to the connecting rod of AB,$\left( {x_{T} ,y_{T} } \right)$ indicates the position of the toe force point $T$, $J$ is the rotational inertia of the talus-shaped parts, and $\ddot{\alpha }_{i}$ is the rotation acceleration of the ankle joint.C. Parameter selections

The value of the prosthetic structure parameter has a great influence on the overall size and driving force of the system, so the optimal value of the structure needs to be obtained through motion mechanics analysis.

**Fig. 11:**
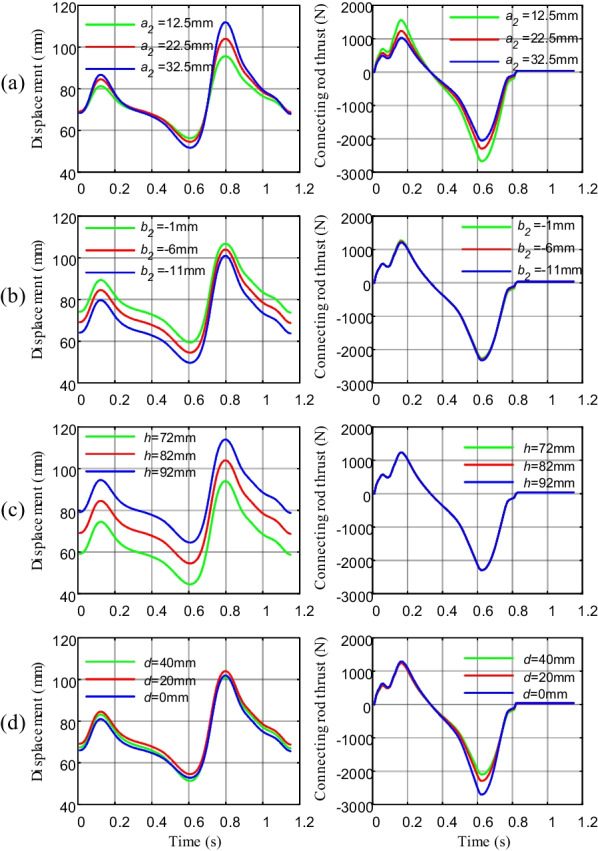
Variation of screw nut displacement and connecting rod thrust. **a** Status of different values of parameter* a*_*2*_. **b** Status of different values of parameter *b*_*2*_. **c** Status of different values of parameter *h*. **d** Status of different values of parameter *d*

Parameters such as (*a*_*2*_*, b*_*2*_*, h, d*) affect the size of the ankle prosthesis and the driving torque. To meet the prosthetic overall dimensional requirements, the initial values of the parameters of the prosthesis are designed according to the structure of the human foot and ankle. Based on the foot–ground contact in the gait support phase, a segmental dynamics model of the prosthetic mechanism was established to achieve the selection of structural parameters optimized by the prosthetic structure size and driving torque. In this section, the variable-controlling approach is used to seek the optimal results of prosthetic structural parameters, which are presented in Fig. 11.

The variation of screw nut displacement and connecting rod thrust is discussed, and the conclusion is deduced as follows. First, there is a positive correlation between the nut displacement and the overall dimensions. As *a*_*2*_ increases, the overall size of the structure increases, and the connecting rod thrust decreases, so the value of *a*_*2*_ should be moderate to minimize the size of the structure without increasing the thrust required to drive the ankle joint. The value of *b*_*2*_ should be reduced as much as possible without causing motion interference, it has little effect on the connecting rod thrust. Reducing the value of h can make the prosthetic system more compact while satisfying the requirements of the range of motion of the mechanism, the effect of h on the thrust of the connecting rod is minimal and negligible. As the value of d increases, the overall range of nut movement becomes smaller, and the required connecting rod thrust decreases. The results of taking values of structural parameters such as (*a*_*2*_*, b*_*2*_*, h, d*) are obtained as shown in Table 3.

**Table 3:** Peak stress on the different thicknesses of prosthetic foot

Parameters	Initial values (mm)	Setting value (mm)
*a*_*2*_	12.5, 22.5, 32.5	22.5
*b*_*2*_	− 1, − 6, − 11	− 11
*h*	72, 82, 92	72
*d*	0, 20, 40	40

### Integration of the ankle–foot prosthesis

**Fig. 12:**
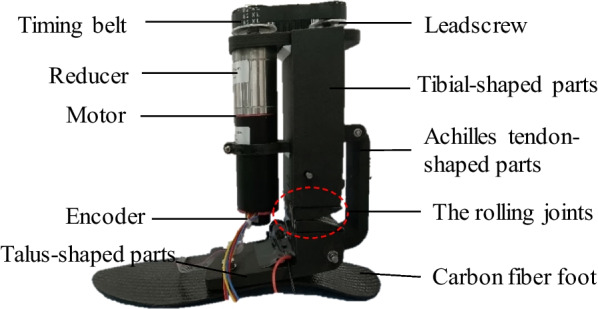
The manufactured prototype of the prosthesis

The manufactured prototype is assembled as shown in Fig. 12. The tibial-shaped parts are embedded with a ball screw sub and support rod, which play the role of connection and support. The screw nut is fixed to the slider, which can drive the up and down movement of the slider by the rotation of the ball screw. The talus-shaped parts are fixedly connected to the energy-storage carbon fiber foot through two threaded holes. They transmit the motion from the Achilles tendon-shaped parts to the joint rolling joints and the energy-storage carbon fiber foot. The missing biological limb imitation function of the proposed ankle–foot prostheses is verified in the next section.

## Experimental validation

**Fig. 13:**
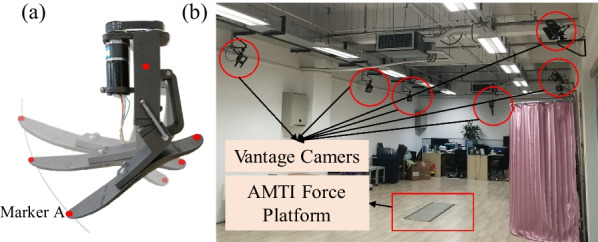
Experimental set-up for the measurements. **a** Attached markers. **b** Experimental setup

The bionic characteristics of the prosthetic joints are verified through the motion detection experiment. We attached markers to the prosthesis and tracked the movements by the motion capture system(VICON) as Fig. 13b presented. The red dots in Fig. 13a indicate the marker positions of the prosthesis mechanism.

### Output trajectory of the ankle–foot prosthesis

**Fig. 14:**
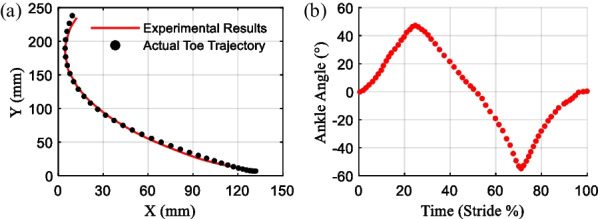
Output trajectory of the ankle–foot prosthesis. **a** The toe trajectory of the prosthesis. **b** The ankle angle of the prosthesis

The marker A represents the actual output point of the toe, its motion was recorded with 10 vantage cameras, which recorded the markers on the structure at a frequency of 100FPS. The marker attached to the tibial-shaped parts was used as the position reference, in the experiment, the motion trajectory of the toe can be obtained by solving the relative position change. The prosthesis transitioned from dorsiflexion to a “zero point state,” subsequently to plantarflexion, and then back to dorsiflexion. In the experiment, the human toe marker point was placed behind the metatarsal bone, whereas the prosthetic toe marker A was affixed to the anterior end of the prosthesis. Since the radii of movement along the conjugate surfaces of the two joints differed, the horizontal and vertical coordinates of the human toe’s movement trajectory were scaled up uniformly during data processing, without altering the trend of the original curve trajectory. The obtained data were compared with the desired trajectory of the output point of the subjects, as shown in Fig. 14a.

It can be seen that the toe of the prosthesis and the human toe movement trajectory fit a high degree, the main error occurs near the maximum dorsiflexion state, the reason is that the slope of the joint surface at this place changes a lot, there is a certain error in the process of parts processing and assembly, but the error is within the allowable range.

The range of the prosthesis motion angle is shown in Fig. 14b. It can be demonstrated that its dorsiflexion angle can reach more than 40° and the plantarflexion angle is close to − 60° in one motion cycle. Its motion range is designed as expected, which is adapted to the mobility of the human joint. The prosthesis has the potential to improve the mobility requirements of lower-limb amputees.

### Energy storage validation

**Fig. 15:**
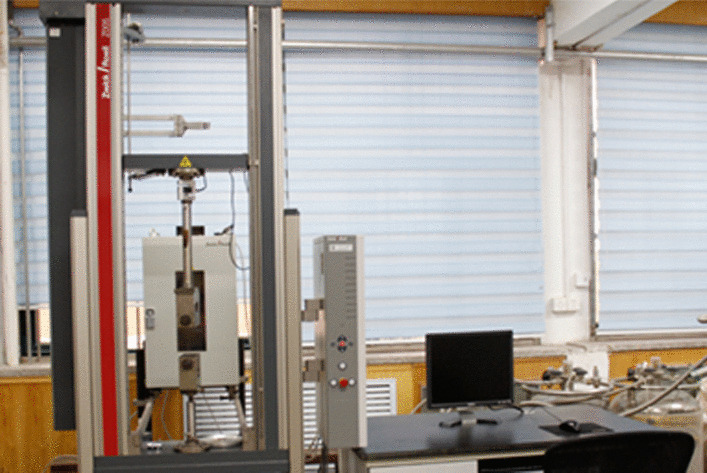
Zwick Z005 Test Bench

We investigated the relationship between the deformation of the carbon fiber energy-storage foot and its elastic strain energy response. The Zwick Z005 pressure platform (Fig. 15) was applied to carry out the verification test, consisting of static and dynamic tests. It was in position control mode during this experiment.

**Fig. 16:**
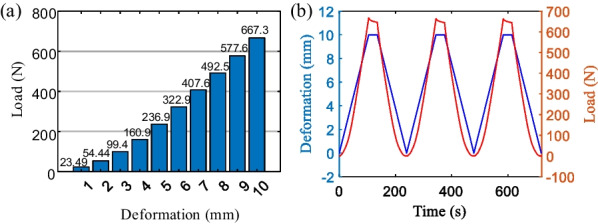
The static pressurization test results

The upper-pressure platform was controlled to descend to the specified position, kept for 30 s, and then returned to the starting position with the same speed. The single experiment ended after three pressures were applied. Since the arch height of the prosthetic foot model is 10 mm, the total amount of position control is set from 1 to 10 mm in 1 mm intervals in the static test respectively. Figure 16a indicates the static pressurization test results, which present the peak load bearing of the carbon fiber energy-storage foot in various deformations.

The static test results are shown in Fig. 16b, in which the position control is set to 10 mm. The blue line indicates the deformation settings and the red one represents the load changes. In the process of maintaining the deformation for 30 s, the load decreases with time and then gradually stabilizes, which is a stress relaxation phenomenon. The load mechanics response process verifies the energy-storage properties of the carbon fiber foot.

**Fig. 17:**
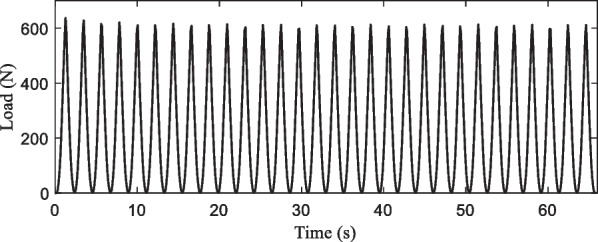
Load response results in the 30 times dynamic test

The foot-flat phase requires a support force size of 600 N, taking the static test results and material creep phenomenon into account, we set the maximum position control target of 9 mm in the dynamic test. Test frequency is required between 0.5 and 3 Hz. In the experiment, it takes 1 s to load to the setting position, and 1 s to return to the starting location, and repeats the above process 30 times to end the single test. Its results are shown in Fig. 17, it can be seen that the 30 times dynamic response results are stable, but there are certain fluctuations in the peak load. Since the error is not monotonically increasing or decreasing, and the absolute value of the error is always within 5%, it can be concluded that there is no plastic deformation of the prosthetic foot during the experiment.

**Fig. 18:**
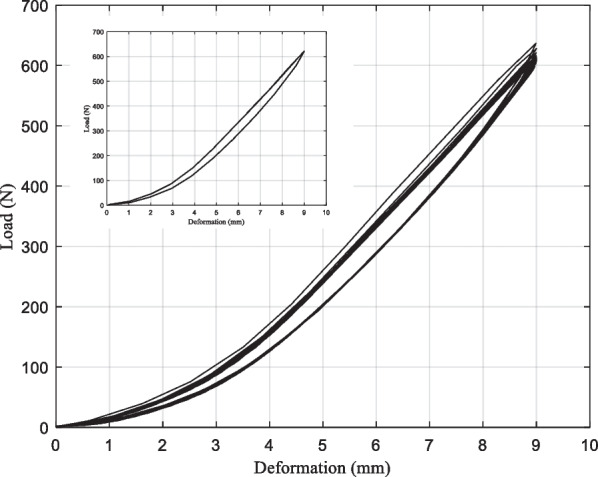
Load-deformation curves of carbon fiber prosthetic feet

The dynamic testing process extracted includes 30 pressurized response curves as shown in Fig. 18. Among them, a single pressurized response curve is presented in the inset figure. Extract the dynamic test process of the single compression response curve, it can be seen when the deformation reaches 9 mm, the size of the resulting stress response is more than 600 N, which conforms to human use requirements. The resulting stress response and the error during the unloading process are less than 45 N. The unloading work corresponds to the release of elastic strain energy in the carbon fiber prosthetic foot during unloading, clearly indicating that the loading work is significantly greater than the unloading work.

**Fig. 19:**
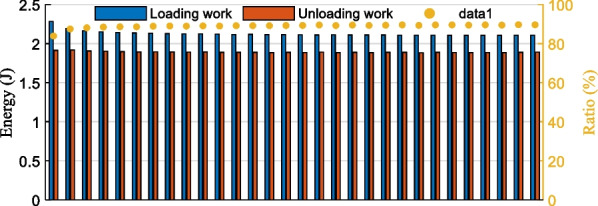
The work of 30 pressurization cycles and the energy released/stored ratio of the carbon fiber foot

Overall, the mechanical response of the prosthetic foot has elastic hysteresis characteristics, which is a common physical phenomenon. It is due to the internal friction of the material resulting in energy dissipation. Statistical the external force work of carbon fiber prosthetic foot in the 30 pressurization cycles dynamic test, its results are shown in Fig. 19. The loading work is the accumulation of external force in the direction of its pressurization, unloading work corresponds to the release of elastic strain energy of the carbon fiber prosthetic foot during the return of the upper-pressure platform, it can be seen that the loading work is significantly greater than the unloading work.

The average value of the energy released/stored ratio of the carbon fiber foot in 30 pressurization cycles was 88.96%. According to the statistical results above, the average value of the elastic strain energy of the carbon fiber prosthetic foot was 1.8912 J when the full foot was set to 9 mm, which indicates that the prosthetic foot can achieve energy storage and release during the movement. It can effectively help to reduce the work done by individuals with below-knee amputation, thus reducing their energy consumption and improving the comfort of wearing the prosthesis.

Traditional ankle–foot prosthetic joints mainly employ fixed-axis rotation and multi-link mechanisms. While fixed-axis rotation meets basic human motion needs, it fails to accurately mimic human ankle joint motion. Multi-link mechanisms, on the other hand, require intricate designs for good fitting but are not suitable for foot and ankle prostheses. Traditional ankle–foot prosthesis comparisons are listed in Table 4.

**Table 4:** Ankle–Foot prosthesis comparison

Structure	Device (Territory)	Features	ROM
Fixed-axis rotating	Pro-Flex Pivot (Ossur)	The poor roll-slip kinematic properties	27°–27°
Four-bar structure	Powered Polycentric Ankle (University of Utah)	Overcomplicated mechanical structure	27°–28°
Four-bar structure	Meridium Ankle (Otto Bock)		N/A
Rolling conjugate surface	Ours	The integration of sliding-rolling technology with energy-storage characteristics	45°–45°

ROM: Plantarflexion-Dorsiflexion; N/A: not applicable

## Conclusion and future work

The proposed design aims to address the limitations of currently available prostheses specifically related to the bionic nature of prosthetic joint motion and energy storage properties of carbon fiber prosthetic foot. In this article, we show how a biomimetic design can enable a single rolling joint to achieve the mixed movement of sliding and rolling while imitating the energy-storage characteristics during the gait cycle.

In addition, the proposed design has a high fitting degree of the trajectory between the prosthetic toe and the human toe. Its range of the proposed ankle joint is greater than 100° (Fig. 14b), meeting the demand for plantarflexion/dorsiflexion motion. Moreover, the experimental results indicated that the proposed powered prosthesis has a mean value of 1.8912 J elastic strain energy, and its energy release/storage ratio of carbon fiber energy-storage foot is 88.96% (Fig. 19), which has the potential to improve the metabolic cost of walking. To this end, we aspire to fully develop functional mobility with the powered ankle–foot prosthesis to satisfy the tradeoff between its functionality and the actuation size and weight. It is helpful to advance a powered ankle–foot prosthesis capable of mimicking human ankle dynamics and to address the unmet requirements of individuals with below-knee amputations.

What’s more, the experimental result shows that the ankle–foot prototype provides suitable movement for typical locomotion tasks, which demonstrates that the proposed design may be a good way to improve comfort and walking economy to fit the prosthesis users.

Finally, future studies should aspire to assess the biomechanical effects of the ankle–foot prosthesis on the residual. We plan to test the proposed powered prosthesis with a lightweight powered knee prosthesis to fit within the anatomical articular structure while providing physiological torque, energy, and range of motion for individuals with above-knee amputations [12, 32]. Meanwhile, to achieve a lightweight yet durable rolling conjugate joint, we utilized the properties of carbon fiber composite materials, integrated them with human joint motion characteristics, and employed topology optimization techniques to accomplish its bionic lightweight upgrading and optimization. Last, but not the least, the replacement of the conjugate rolling joint module is expected to enable individualized prosthetic design for lower limb amputees and their clinical rehabilitation needs.

## Data Availability

Any material in this paper that is the original work of the authors.All of the material is owned by the authors and no permissions are required. The data that support the findings of this study are available from the corresponding author upon reasonable request.
